# The Relationship Between the Interpersonal Relationship and Altruistic Behavior of College Students Majoring in Physical Education: The Mediating Effect of Empathy and the Moderating Effect of Responsibility

**DOI:** 10.3390/bs14121240

**Published:** 2024-12-23

**Authors:** Tuojian Li, Wanxuan Feng, Hongzhen Zhang

**Affiliations:** School of Physical Education, Shandong University, Jingshi Road, Lixia District, Jinan 250014, China; lituojian@sdu.edu.cn (T.L.); fengwanxuan@mail.sdu.edu.cn (W.F.)

**Keywords:** interpersonal relationship, empathy, social responsibility, altruistic behavior, sports major students

## Abstract

Students majoring in physical education are the main source of physical education teachers in Chinese schools, and they are the main body in achieving the function of physical education in schools. The improvement of their altruistic behavior helps enhance the educational literacy of outstanding physical education teachers. This study conducted a questionnaire survey on 310 students majoring in physical education from 5 universities in 5 selected regions using the General Interpersonal Communication Objective Scale, Interpersonal Reactivity Index-C, the “responsibility” subscale of the Big Five Inventory Questionnaire, and the Altruistic Behavior of College Students questionnaire. We performed correlation analysis, mediation effect analysis, and moderation effect analysis on the data using SPSS 24.0 and MPLUS 7.4. Empathy partially mediates 30% of the total effect between interpersonal relationships and altruistic behavior; social responsibility has a moderating effect on empathy and altruistic behavior, with higher levels of responsibility having a greater moderating effect. Interpersonal relationships, empathy, and a sense of responsibility affect the current, recent, and long-term states of altruistic behavior, respectively. To improve students’ altruistic behavior throughout the entire process, schools should develop training systems specifically for interpersonal relationships, empathy, and social responsibility.

## 1. Introduction

Altruism is one of the important values for physical education teachers in the teaching process. Under the guidance of altruistic values, physical education teachers can better educate and protect students. Altruistic behavior can be classified as pro-social tendencies or behaviors that are beneficial or helpful to others [1].The pre-service and school stage is a crucial period for physical education teachers to improve their comprehensive literacy, cultivate their sense of social responsibility, and cultivate altruistic behavior [2,3]. Students majoring in physical education are the main source of physical education teachers in China and the main body in achieving the function of physical education in schools. However, their socialist core values, disconnected knowledge and action, and weak altruistic behavior and concepts can cause the social responsibility of the physical education teacher group to be dispersed and the social value system to be shaken [4]. Exploring the predictive factors and improvement mechanisms of altruistic behavior among college students majoring in physical education can provide a theoretical basis and practical guidance for the targeted prediction and improvement of altruistic behavior among physical education teacher talent training units, promote the psychological health development of students [5], integrate altruistic education into pre- and post-service physical education teachers, and realize the implicit educational function of school physical education. At present, the relationship between interpersonal relationships, altruistic behavior, empathy, and social responsibility in the field of sports has not been fully studied. This study further explored the correlation between these variables and filled the gaps in previous research.

### 1.1. Interpersonal Relationships, Empathy, and Altruistic Behavior

Altruism is a behavioral tendency or reaction, specifically referring to the behavior of individuals giving up more of their own rewards to help others in high-cost situations [6]. The predictive and influential role of interpersonal relationships in altruistic behavior has been confirmed in many empirical studies. Interpersonal relationships refer to the emotional connections formed between individuals through direct communication, which can bring satisfying feelings to individuals spiritually and emotionally, and are their basic psychological needs. Some studies have shown that individual altruistic behavior is influenced by interpersonal responsibility, type of interpersonal relationship, orientation of interpersonal relationship, closeness of interpersonal relationship itself, or social distance between individuals [7]. In addition, altruistic behavior is significantly associated with empathy [8], and according to the empathy altruism hypothesis, the feeling or emotion of empathy is the key to triggering the motivation for altruistic behavior [9]. Empathy mainly includes three aspects, one of which is that the observer’s feelings must match those of the observed. Secondly, the observer’s perception is only adapted to the other person’s emotional state in other ways. Thirdly, the observer’s feelings must be concern or sympathy for the pain of others [10]. In an empirical study, it was clearly pointed out that there is a positive and significant correlation between altruism and empathy [11]. The expression of empathy and altruistic behavior among individuals varies, and it is influenced by many factors, such as an individual’s emotional state, initiation scenario, etc. [12]. Empathy and interpersonal relationships are also closely related. Studies have shown that empathy is an effective predictor of interpersonal relationships, but empathy ability is not positively correlated with interpersonal relationships. Higher levels of empathy ability can limit the development of interpersonal relationships [13]. Jordan pointed out in his research that the correlation between empathy and interpersonal relationships varies depending on the subject of the interpersonal relationship [14].

Interpersonal relationship ability is easy to capture and evaluate in the daily behavior of sports major students. Using student interpersonal relationships as a starting point to identify individuals that require special attention can effectively improve prediction efficiency. But so far, it has not been confirmed whether different interpersonal communication goals have different predictive effects on altruistic behavior. Empathy, as an indispensable prerequisite for studying altruistic behavior, is closely related to altruism and interpersonal relationships. The specific relationship and mechanism of action between the three variables are still unclear. Therefore, this study investigates and explores the mechanisms of the three as a complete system, clarifying whether interpersonal relationships are effective predictors of empathy and altruistic behavior, verifying the mediating role and magnitude of empathy. The final fitting efficiency of the three-element model determines the efficiency of interpersonal relationships in predicting altruistic behavior, which becomes the first question of this study.

### 1.2. Empathy, Altruistic Behavior, and Social Responsibility

Altruistic behavior is influenced by the heterogeneity of individual personality factors, among which the impact of individual social responsibility on altruistic behavior is the focus of many studies [15]. Social responsibility refers to the recognition, emotion, and behavior of social groups or individuals toward the responsibilities and obligations they undertake in their corresponding social roles [16]. It is a positive and altruistic psychological quality. Research has shown that social responsibility is the determining factor of altruistic behavior [17], and the higher the level of social responsibility, the higher the level of altruistic behavior [18]. In addition, studies have shown that the sense of responsibility has cross-situational consistency and an individual’s sense of responsibility in one context (such as family) have a strong predictive effect on their sense of responsibility in another context [19]. There is a significant positive correlation between empathy and social responsibility [20], and research suggests that the improvement of social responsibility level lies in the increase in empathy ability and emotional regulation level [16]. Research suggests that in the context of the COVID-19 pandemic, the social responsibility of college students to some extent mediates the relationship between empathy and prosocial behavior [21]. Zheng Xianliang (2017) explored the intrinsic mechanism of the relationship between network altruistic behavior and adolescent subjective well-being under the regulatory effect of a sense of responsibility [22]. Wang Wan (2018) also chose social responsibility as a moderating variable to explain the impact of perceived anonymity on third-party punishment on the internet [23].

Overall, social responsibility is correlated with altruistic behavior and empathy, but it is unclear what role social responsibility plays in mediating the influence of empathy on altruistic behavior in interpersonal relationships. Therefore, this study used social responsibility as a moderating variable to examine whether the level of social responsibility has different effects on interpersonal relationships, empathy, and altruistic behavior. This clarifies the second research question of this study, which is whether the level of social responsibility will have different moderating effects on the relationship between interpersonal relationships and altruistic behavior.

Although there are many studies on altruistic behavior and interpersonal relationships, research in the field of sports is still immature. Altruistic behavior plays an important role in physical education, and it is necessary for us to further study its intrinsic connection. Therefore, this study focuses on students majoring in physical education as the survey subjects, investigates their interpersonal relationships, empathy, altruistic behavior, and social responsibility, constructs a model of altruistic behavior among students majoring in physical education, and proposes the following research hypotheses:

**H1.**  *Interpersonal relationships can effectively predict altruistic behavior, and empathy plays a certain mediating role between the two.*

**H2.**  *Social responsibility is a moderating variable of altruistic behavior, which has a certain moderating effect on interpersonal relationships, empathy, and altruistic behavior.*

## 2. Method

### 2.1. Design

This study is a cross-sectional study aimed at investigating the relationship between interpersonal relationships, empathy, altruistic behavior, and social responsibility among participants. All participants signed informed consent before the start of the research. They were informed of the procedure to be followed, as well as the questionnaires to be completed, and the aim pursued with the research. They were also informed that the treatment of the data would be completely anonymous and that only the project researchers would use the data. Participation was voluntary, and the participants were informed that they could withdraw from the research at any time, which would result in the elimination of their data collected up to that point. All experiments in this study were conducted in accordance with the Helsinki Declaration of Ethical Principles for Human Medical Research and other relevant laws, regulations, and ethical norms.

### 2.2. Participants

G*Power 3.1^TM^ was used to calculate the sample size in this study. The program used four predictor variables (interpersonal relationships, empathy, altruistic behavior, and social responsibility) as the statistical basis for linear multiple-regression analysis (linear multiple regression: fixed model, R^2^ deviation from zero). Therefore, the study used effect values of power (1 − β) = 0.95, alpha = 0.05, and f^2^ = 0.15 (with a moderate effect size of 0.15) as specific parameters to calculate the corresponding sample size. Finally, it was determined that the study requires at least 129 participants to ensure its scientific validity.

The data in this study were obtained through convenient sampling surveys. According to the list of Chinese universities, the survey questionnaire was distributed to sports majors from five universities in Beijing, Shanghai, Guangzhou, Fujian, and Shandong from October to December 2023, with a total of 340 questionnaires distributed.

All participants had to meet the following inclusion criteria: (a) age between 18 and 22 years; (b) college students majoring in sports; (c) voluntary participation in research; (d) there were no disabilities or illnesses that hindered the completion of the questionnaire. The exclusion criterion was (a) an incomplete completion of all the questionnaires.

Ultimately, we collected 310 valid questionnaires with an effective response rate of 91.17%. According to statistical analysis, these responses came from 168 males and 142 females with an average age of 20.07 ± 1.38 years.

### 2.3. Measures

According to the proposed hypotheses, there are four variables—interpersonal relationship, empathy, social responsibility, and altruistic behavior—that should be tested in the investigation. Referring to the previous related papers, a questionnaire including 74 items collected from the corresponding four parts was designed to test the proposed hypotheses.

#### 2.3.1. Interpersonal Relationship

Interpersonal relationships were measured using the General Interpersonal Communication Objectives Scale developed by Canevello and Crocker (2010) and revised by Zhang Min, Zhang Lin, and Corcker (2012) [24]. This scale consists of 18 items and is scored on a 5-point scale, ranging from 1 (strongly disagree) to 5 (strongly agree). The higher the score, the better the interpersonal relationship. Previous studies have shown that the scale has good reliability and validity. In this study, Cronbach’s alpha of the questionnaire was 0.931.

#### 2.3.2. Empathy

The measurement of empathy was conducted using the Chinese version of the scale developed by Davis (1983) and revised by Fengfeng Zhang et al., which measures the stable empathy ability of participants in their daily lives [25]. This stable empathy can be seen as an individual’s personality traits or general abilities, namely trait empathy [26]. This scale includes four dimensions: viewpoint selection, imagination, empathy concern, and personal pain, totaling 22 items. Among them, viewpoint selection and imagination are considered cognitive empathy, while sympathy, attention, and personal pain are considered emotional empathy. It uses a 5-point scoring system, ranging from 0 (strongly disagree) to 4 (strongly agree). The higher the score, the higher the level of empathy. This scale has been proven to have good reliability and validity in previous studies [27]. In this study, Cronbach’s alpha of the questionnaire was 0.734.

#### 2.3.3. Social Responsibility

The sense of social responsibility was measured using the “conscientiousness” subscale developed by John et al. in the Big Five Inventory (BFI) [28]. This subscale consists of 12 items and uses a 5-point scoring system, ranging from 1 (strongly disagree) to 5 (strongly agree). The higher the score, the higher the sense of social responsibility. This scale is currently the most widely used scale both domestically and internationally, and is a commonly used tool for measuring a sense of responsibility, with good reliability [29]. In this study, the α coefficient was 0.928.

#### 2.3.4. Altruistic Behavior

The Altruistic Behavior of College Students questionnaire consists of 22 questions, including five dimensions: altruistic responsibility, respect and care for others, care and concern for oneself, altruistic behavior performance, selfish behavior, and concept [30]. The questionnaire adopts a 7-point scoring system from strongly agree to strongly disagree, with a higher total score indicating a higher level of altruism. This questionnaire is used with an α coefficient of 0.738.

### 2.4. Procedure

These data were collected through electronic questionnaires distributed to participants on online platforms, and were authorized by relevant teachers of the school before conducting the survey. Each school sent approximately 68 questionnaires, depending on the number of students participating in the survey. Before filling out the questionnaire, each participant received informed consent form, and the questionnaire also requires each participant to fill out the questionnaire separately. To ensure a high response rate to the questionnaire, each student who completes the questionnaire is required to send a screenshot to the researchers.

### 2.5. Data Analysis

Common method bias can occur since the dataset collected by the present study was obtained from the measurement of four variables, including interpersonal relationship, empathy, altruistic behavior, and social responsibility through the self-report method. Therefore, the procedure method was adopted by the present study to control the bias, and the proposed procedures were as follows: (1) each part of the questionnaire was selected from a mature questionnaire with high reliability and validity, that has been used both domestically and internationally to reduce the systematic errors in measurement. (2) The questionnaire survey was completely anonymous so that the respondents could answer the questions according to their real situations. (3) Reverse scoring was used for some of the items in the questionnaire. (4) The items were arranged in different order in two versions of the questionnaire to avoid the possible errors caused by the order of these items. After the data collection, the Harmans single-factor test was used to test the common method bias of the obtained dataset, and the results revealed that the first factor explained the variation of 19.88%, which is less than half of the total variation explained (67.73%) and less than the cut-off criterion of 40%. The common method bias of the present study is not significant.

We centralized all data using SPSS 24.0, converted them into Z-scores, and tested multicollinearity. Next, MPLUS7.4 was used to test the mediating effect of empathy between interpersonal relationships and altruistic behavior, with interpersonal relationships as the independent variable, empathy as the mediating variable, and altruistic behavior as the dependent variable. We set the number of Bootstrap iterations to 5000 and used the bias-corrected percentile Bootstrap method to estimate the significance of specific mediating effects.

## 3. Results

### 3.1. Association Analysis of Interpersonal Relationship, Empathy, Social Responsibility, and Altruistic Behavior

Firstly, we calculated the mean and standard deviation of the four variables, and then conducted correlation analysis. As shown in Table 1, there is a significant positive correlation between interpersonal relationships, empathy, altruistic behavior, and social responsibility.

### 3.2. The Mediating Effect of Empathy in Interpersonal Relationships and Altruistic Behavior

From the analysis results, it can be seen that there is a significant positive correlation between interpersonal relationships, empathy, social responsibility, and altruistic behavior. However, the relationship between each element is not clear, suggesting a more complex relationship between interpersonal relationships, empathy, social responsibility, and altruistic behavior. To analyze the mechanism through which interpersonal relationships influence altruistic behavior, we further examined the mediating effect of empathy between interpersonal relationships and altruistic behavior.

Then, using interpersonal relationship as the independent variable, empathy as the mediating variable, and altruistic behavior as the dependent variable, the mediating effect of empathy between the other two variables was tested by the software MPLUS7.4. From Table 2, it can be seen that interpersonal relationships have a significant positive impact on empathy, with a confidence interval of 0.337 (lower bound = 0.262, upper bound = 0.337), excluding zeros within the interval. From Table 3, it can be seen that interpersonal relationships have a significant positive impact on altruistic behavior, with a confidence interval of 0.353 (lower bound = 0.213, upper bound = 0.353), excluding zeros within the interval. Empathy has a significant positive impact on altruistic behavior, with a confidence interval of 0.399 (lower bound = 0.22, upper bound = 0.399), excluding zeros within the interval. From Table 4, it can be seen that the indirect effect was significant with a confidence interval (lower bound = 0.074, upper bound = 0.135), excluding zeros within the interval. The size of the indirect effect was 0.135. The overall effect was significant, with a confidence interval (lower bound = 0.363, upper bound = 0.488), excluding zeros within the interval. The size of the total effect was 0.488.

**Figure 1:**
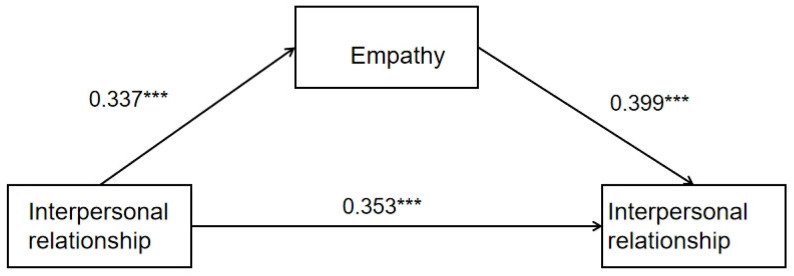
Empathy mediation model of interpersonal relationship and altruistic behavior. *** *p* < 0.001.

As a result, the relationship of the three variables was clarified and H1 was verified. The interpersonal relationship can effectively predict altruistic behavior, where empathy plays a partly mediating role between the two variables. As shown in Figure 1, an empathy mediation model of interpersonal relationship and altruistic behavior can be established based on the above results.

### 3.3. The Relationship Between Interpersonal Relationships and Altruistic Behavior: Moderating Effect Test of Empathy and Social Responsibility as Mediators

As the mediating effect of empathy has been verified in the previous section, combining the related theory from previous studies, the relationship between interpersonal relationship, empathy, and altruistic behavior is possible to moderate by social responsibility, which means that a moderated mediation model is possibly established to discriminate the relationship between interpersonal relationship, altruistic behavior, empathy, and social responsibility. SPSS 24.0 was used for the multicollinearity test of the four variables. The value of tolerance was higher than 0.9, the VIF was lower than 1.3, and the condition index was lower than 1.47 for each variable. This result indicates that there is no multicollinearity issue among the four variables, and the selected four variables are completely independent without any linear or highly correlated relationship.

This article used MPLUS7.4 to test the mediating moderating effect, with interpersonal relationships as the independent variable, social responsibility as the moderating variable, empathy as the mediating variable, and altruistic behavior as the dependent variable. The result of regression analysis on empathy is shown in Table 5. Obviously, there was a significant positive effect of interpersonal relationship on empathy with an acceptable confidence interval (lower bound = 0.193, upper bound = 0.314), excluding zero in the interval, and the coefficient was 0.251. The effect of social responsibility on empathy was also significant positive with an acceptable confidence interval (lower bound = 0.159, upper bound = 0.319), excluding zero in the interval, and the coefficient was 0.243. Additionally, the effect of the interaction term of interpersonal relationship and social responsibility on empathy was also significant positive with an acceptable confidence interval (lower bound = 0.009, upper bound = 0.019), excluding zero in the interval, and the coefficient was 0.243, which means that social responsibility has a positive moderating effect on empathy.

The result of regression analysis on altruistic behavior is shown in Table 6. Obviously, there was a significant positive effect of interpersonal relationship on altruistic behavior with an acceptable confidence interval (lower bound = 0.221, upper bound = 0.333), excluding zero in the interval, and the coefficient was 0.333. The effect of empathy on altruistic behavior was also significantly positive, with an acceptable confidence interval (lower bound = 0.091, upper bound = 0.256), excluding zero in the interval, and the coefficient was 0.256. Alongside that, the effect of the interaction term of interpersonal relationship and social responsibility on altruistic behavior was also significant positive with an acceptable confidence interval (lower bound = 0.003, upper bound = 0.013), excluding zero in the interval, and the coefficient was 0.013, which means that social responsibility has a positive moderating effect on altruistic behavior.

The direct and indirect moderating effects of social responsibility were not fixed, and they could be different if the value of the social responsibility changed. In the present study, the obtained data were bound by one standard deviation above and below the mean values of social responsibility, and divided into three groups: the low-value group, medium-value group, and high-value group. As shown in Table 7, the confidence intervals of the direct effect, indirect effect, and total effect for the three groups did not contain the value of zero, which means that the moderating effects of social responsibility for all the groups are significant.

According to the data shown in Table 7, the moderating effect through the mediating path of empathy is relatively small, while the indirect regulatory effect of the low-value group is 0.026 (95% CI = [0.005, 0.069]). The indirect moderating effect of the median group is 0.064 (95% CI = [0.023, 0.116]). The indirect regulatory effect is 0.102 for the high-value group (95% CI = [0.036, 0.18]). The direct moderating effect of social responsibility on interpersonal relationships and altruistic behavior in the three groups is as follows: the low-value group has a direct moderating effect of 0.202 (95% CI = [0.049, 0.363]); the median group had a direct moderating effect of 0.333 (95% CI = [0.221, 0.441]); and the direct regulatory effect of the high-value group is 0.463 (95% CI = [0.33, 0.61]). The overall moderating effect of a sense of responsibility on interpersonal relationships and altruistic behavior had values for the low, medium, and high groups of 0.228 for the low-value group and a confidence interval of (lower bound = 0.075, upper bound = 0.397). The median group is 0.397, with a confidence interval of (lower bound = 0.286, upper bound = 0.504). The confidence interval of 0.565 for the high-value group is (lower bound = 0.437, upper bound = 0.688).

The confidence interval for the indirect moderating effect of the interaction term between social responsibility and interpersonal relationships through the empathy mediation pathway on interpersonal relationships and altruistic behavior is (lower bound = 0.001, upper bound = 0.007), and the interval does not include zero, indicating that the moderating effect of the interaction term through empathy was significant, with a moderating effect size of 0.004. Please refer to Table 8 for details.

**Figure 2:**
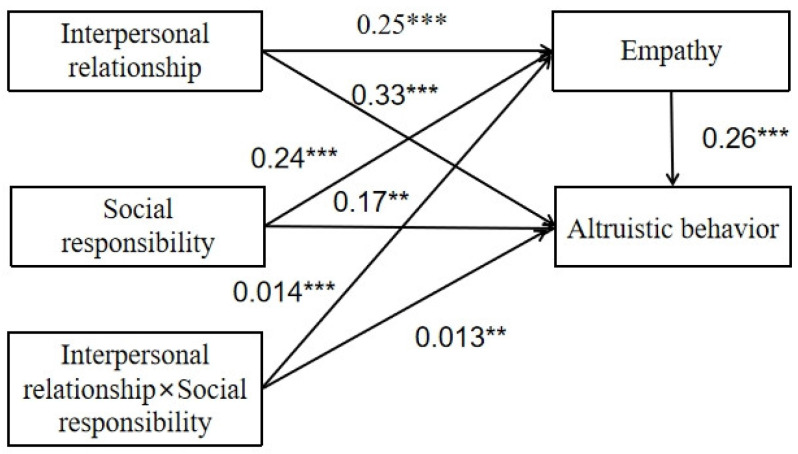
The social responsibility adjustment model of interpersonal relations and altruistic behavior under empathy mediation. *** *p* < 0.001, ** *p* < 0.01.

Overall, social responsibility is a moderating variable for altruistic behavior, and the moderating effect was significant for interpersonal relationship, empathy, and altruistic behavior. The hypothesis of H2 was verified, and a moderated mediation model can be established using social responsibility as the moderating variable, empathy as mediating variable, interpersonal relationship as the independent variable, and altruistic behavior as the dependent variable. The relationship between the variables and the related coefficient for each path are shown in Figure 2.

### 3.4. Johnson–Neyman Test for the Moderating Effect of Social Responsibility

The results in the previous section revealed that the moderating effect of social responsibility on interpersonal relationship and altruistic behavior was mainly reliant on the direct path and the first half of the indirect mediating path. In order to identify how the factor of social responsibility moderates, the present study analyzed the moderating mechanisms, confidence intervals, and Johnson–Neyman intervals of the two paths.

**Figure 3:**
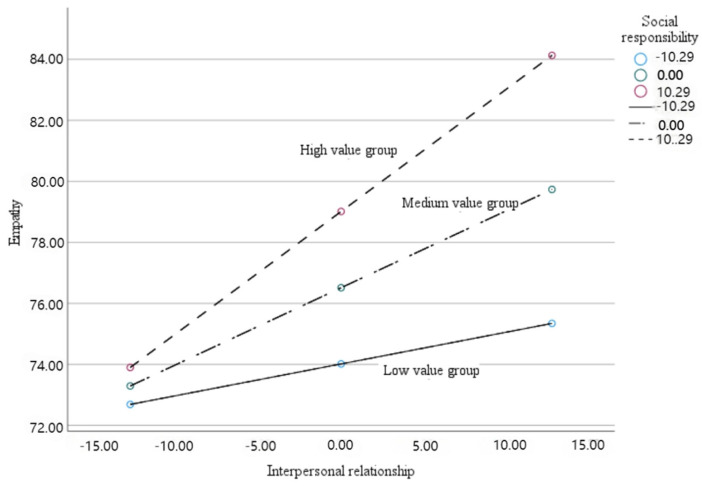
Regression comparison of interpersonal relationships and empathy among high-, medium-, and low-value responsibility groups.

Firstly, the moderating effect of social responsibility on the indirect path is significant, where empathy plays a mediating role between interpersonal relationship and altruistic behavior. Next, the obtained data was divided into three groups including low value group, medium value group and high value group, using the one standard deviation above and below the mean values of social responsibility as the boundary points. As shown in Figure 3, the regression coefficients of empathy on altruistic behavior were all significant (*p* < 0.05) for all the three groups, which means that the better the interpersonal relationship, the higher the empathy ability. Furthermore, it is obvious that slope of the regression line for the high-value group is higher than the low-value group, suggesting that the moderating effect of social responsibility is higher in the high-value group than in the low-value group since the empathy varies more with changes in interpersonal relationship.

**Figure 4:**
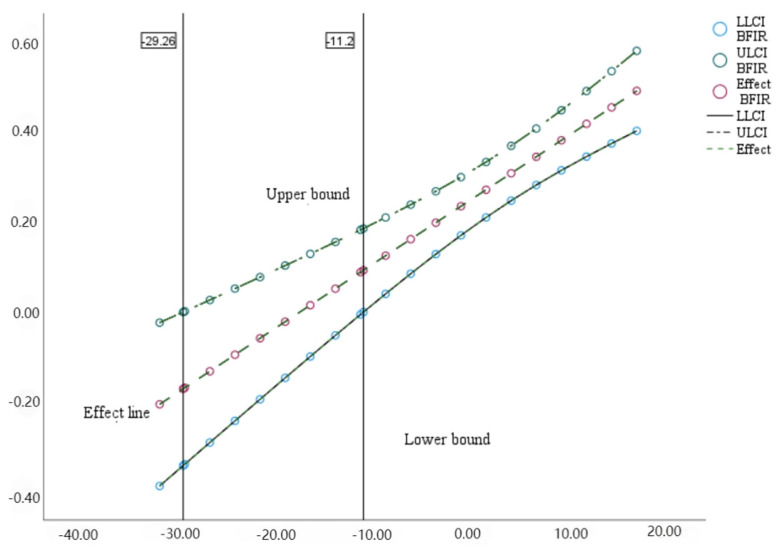
Confidence interval and the Johnson–Neyman point of the moderating effect of a sense of responsibility on interpersonal relationships and empathy.

The Johnson–Neyman method was used in the next step to identify the confidence interval and significant interval of the moderating effect of social responsibility on the interaction of interpersonal relationship and empathy. As shown in Figure 4, when the value of social responsibility is lower than −29.26 (Approx. Mean-2.5SD) or higher than −11.2 (Approx. Mean-1SD), the moderating effect of social responsibility is significant for the indirect path in statistics.

**Figure 5:**
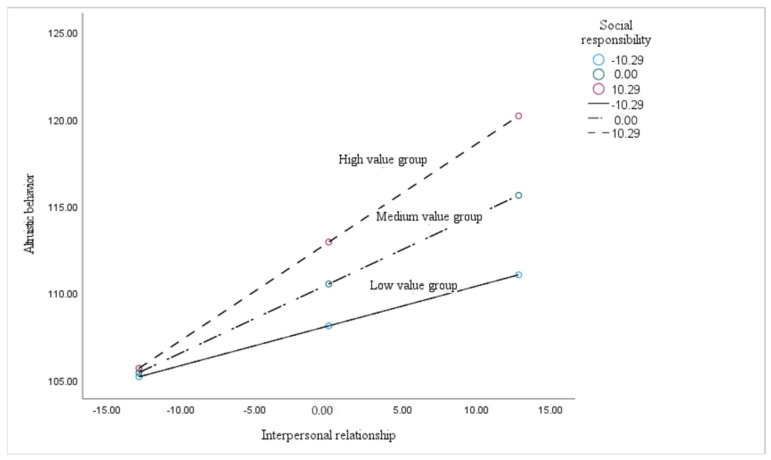
Regression comparison of interpersonal relationships and altruistic behavior among high-, medium-, and low-value responsibility groups.

**Figure 6:**
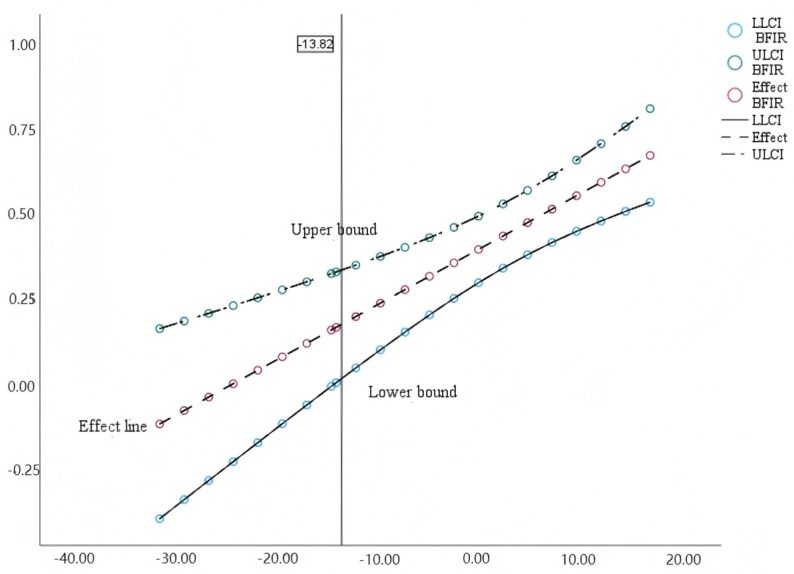
Confidence interval and the Johnson–Neyman point of the moderating effect of the sense of responsibility group on interpersonal relationships and altruistic behavior.

As mentioned in the previous section, the moderating effect of social responsibility is also significant for the direct path between interpersonal relationship and altruistic behavior. The dataset was also divided into three groups, including the high-value group, medium-value group, and low-value group, as mentioned before. As shown in Figure 5, the regression coefficients of interpersonal relationship on altruistic are all significant (*p* < 0.05) for all three groups, which means that the better the interpersonal relationship, the higher possibility the altruistic behavior. The slope of the regression line for the high-value group was also higher than the low-value group, suggesting that the moderating effect of social responsibility is stronger in the high-value group than in the low-value group. Furthermore, the method of Johnson–Neyman was used to identify whether the moderating effect of social responsibility on the direct path was statistically significant only when the value of social responsibility was higher than −13.82 (approx. mean-1SD).The result is shown in Figure 6.

## 4. Discussions

### 4.1. Empathy Plays a Partial Mediating Role Between Interpersonal Relationships and Altruistic Behavior

This study investigated the effects of interpersonal relationships, empathy, and social responsibility on altruistic behavior among sports major college students. The results showed a significant positive correlation among the four factors, which is consistent with previous research findings and confirms that interpersonal relationships can effectively predict empathy and altruistic behavior [31]. The pairwise association of interpersonal relationship, empathy, and altruistic behavior provided the present study a basis for analyzing the mediating effect of empathy between interpersonal relationship and altruistic behavior. According to the results, the mediating effect of empathy accounts for 27.66% of the total effects between interpersonal relationship and altruistic behavior. That is to say, the altruistic behavior is affected by interpersonal relationships through two paths. The first path is a direct path that interpersonal relationships can directly affect altruistic behavior through. The second path is an indirect path, where interpersonal relationships can affect altruistic behavior through the mediation of empathy, and the effect of the indirect path accounts for almost one-third of the overall effect. The direct effect and indirect effect of interpersonal relationship are both positive, which means that an individual with a higher interpersonal relationship will show better performance for empathy and altruistic behavior. In other words, interpersonal relationship is a valid predictor of empathy and altruistic behavior. Using the proposed mediating model, it is possible to initially predict an individual’s empathy ability and altruistic behavior based on their performance related to interpersonal relationships.

### 4.2. Social Responsibility Plays an Important Regulatory Role in Interpersonal Relationships and Altruistic Behavior

The moderating effect of social responsibility on the proposed mediating model is also verified, and the variable of social responsibility plays a moderating role in both the direct path and indirect path. Specifically, the better the interpersonal relationship, the better the empathy ability and the higher the possibility of altruistic behavior in all the three groups divided by value of social responsibility. The path coefficients of the moderating effect of social responsibility on empathy and altruistic behavior were all significantly positive. Social responsibility is derived from an individual’s sense of social values and behavior norms and is expressed through a correct understanding of responsibilities and correct behaviors, such as helping others. The results that social responsibility can somehow predict an individual’s altruistic behavior are also consistent with the previous related studies [18,32,33,34].

However, although the moderating effect by social responsibility is positive for both the direct and indirect path, the path coefficient was only 0.014 for the direct one and 0.013 for the indirect one. One of the reasons estimated is that altruistic behavior is not the same with altruism, which means that persons with altruistic intentions may not put their intentions into practice. An investigation of altruistic behavior should not ignore the cost of helping others [35]. The implementation of altruistic behavior is a cognitive-based decision-making process, and the incentive theory holds that persons will make a cognitive decision-making process according to the logic of “loss-reward” before implementing altruistic behavior. Whether the final altruistic behavior is implemented is based on the measurement of loss or reward that the behavior will bring; meanwhile, the decision-making process of altruistic behavior may also be influenced by kinds of factors and complex mechanisms that make it hard to predict. Moreover, the composition of social responsibility is also complicate, and there are many different manifestations such as “do good”, “not do bad” and “correcting mistakes”, which can be easily affected by environmental factors. A little change may lead to a huge behavioral response gap. In conclusion, the moderating effect of social responsibility is affected by reasons from both altruistic behavior and social responsibility, resulting in two small path coefficients for the direct and indirect paths.

### 4.3. There Is a Mediating Regulatory Model Between Interpersonal Relationships and Altruistic Behavior

On the basis of the mediating effect of empathy and the moderating effect of sense of responsibility, a systematic analysis was conducted on the internal mechanism of interpersonal relationships affecting altruistic behavior. We verified the existence of a mediating moderating model in the relationship between interpersonal relationships and altruistic behavior [36]. Specifically, empathy plays a partial mediating role between interpersonal relationship and altruistic behavior, while social responsibility moderates the first half of the indirect effect and the direct effect of the mediating model. This is a further detailed conclusion about the relationship between interpersonal relationship and altruistic behavior on the basis of previous studies.

Based on the analysis of the moderated mediation model, it was found that the generation of altruistic behavior is a process of complex psychological activities during which a decision is made based on these psychological analyses. It is possible to predict altruistic behavior by interpersonal relationship, where empathy plays an important mediating role and social responsibility a moderating role. Social responsibility is a stable inner-personality trait which cannot only moderate an individual’s altruistic behavior, but also affect one’s behavior in the long term.

In summary, rather than an instantaneous behavioral decision, the implementation and maintenance of altruistic behavior is more of an overall behavioral tendency in time series composed of multiple instantaneous decisions. It is possible that interpersonal relationship, empathy, and social responsibility affect the current state, recent state, and long-term state of altruistic behavior, respectively. The formulation of altruistic behavior cannot be simply achieved at one stroke, but the result of a combination of factors. However, the present study just finds the moderating effect of social responsibility in the mediating model, but the moderating mechanism is still not clear. There are still problems of unclear conception, connotation, and extension in the study of altruistic behavior to be resolved in the future, such as pathological altruistic factors, which are difficult to strip away, and altruistic and altruistic behavior difficulty in discriminating.

### 4.4. Limitations

The limitations of this study mainly include the following points. Firstly, the data obtained were collected through a self-assessment questionnaire survey of college students, which was based on subjective evaluation. In the future, new methods such as self-assessment reports and other evaluations will be adopted to improve objectivity. Secondly, as cross-sectional analysis cannot be validated in a time series, it is believed that future tracking and follow-up investigations would be better. Thirdly, the sample only includes China, which is not conducive to the generalization of the results. Fourthly, this study aimed to elucidate the relationship between empathy and interpersonal relationships, altruism, and social responsibility. Therefore, it did not explore empathy and altruistic behavior from multiple dimensions. In future research, the role of multiple dimensions of empathy and altruistic behavior will be further explored.

## 5. Conclusions

The mechanism presented in the proposed moderated mediating model revealed how altruistic behavior is affected by interpersonal relationship, which is meaningful for improving the altruistic behavior of college students majoring in physical education. Firstly, altruistic behavior is a behavioral tendency in a time series composed of multiple instantaneous decisions. The cultivation of altruistic behavior is also a long-term process. Since interpersonal relationship, empathy, and social responsibility affect the current state, recent state, and long-term state of altruistic behavior, respectively, it is important to make an education plan for improving interpersonal relationship, empathy, and social responsibility. Secondly, interpersonal relationship is a factor that can be identified and evaluated easily in ordinary life of college students, and it is believed to be efficient to improve altruistic behavior through the evaluation and improvement of interpersonal relationship. Thirdly, it is also important for empathy training since the mediating role it played in the correlation between interpersonal relationship and altruistic behavior. Lastly, the education of responsibility should also be included in the physical education system due to the moderating effect of social responsibility on empathy and altruistic behavior.

## Figures and Tables

**Table 1:** An analysis of the association between interpersonal relationships, empathy, social responsibility, and altruistic behavior.

Variable	M	SD	Interpersonal Relationship	Social Responsibility	Emotional Empathy	Cognitive Empathy	Altruistic Behavior
interpersonal relationship	93.32	12.85	1				
social responsibility	43.63	10.29	0.233 **	1			
emotional empathy	37.05	4.92	0.297 **	0.293 **	1		
cognitive empathy	39.78	4.75	0.526 **	0.327 **	0.439 **	1	
altruistic behavior	111.01	12.57	0.498 **	0.278 **	0.244 **	0.500 **	1
empathy	76.95	8.55	0.507 **	0.233 **	0.844 **	0.832 **	0.454 **

** *p* < 0.01.

**Table 2:** Regression analysis of empathy in mediation effect.

	Dependent Variable: Empathy					
Independent Variable	Estimate	S.E.	Est./S.E.	*p*-Value	Lower Bound	Upper Bound
Interpersonal relationship	0.337	0.038	8.862	0.000	0.262	0.337

**Table 3:** Regression analysis of altruistic behaviors in mediation effect.

	Dependent Variable: Altruistic Behaviors					
Independent Variable	Estimate	S.E.	Est./S.E.	*p*-Value	Lower Bound	Upper Bound
Interpersonal relationship	0.353	0.070	5.081	0.000	0.213	0.353
Empathy	0.399	0.092	4.348	0.000	0.22	0.399

**Table 4:** Mediating effects.

	Estimate	S.E.	Est./S.E.	*p*-Value	Lower Bound	Upper Bound
Indirect effect	0.135	0.035	3.892	0.000	0.074	0.135
Total effect	0.488	0.062	7.871	0.000	0.363	0.488

**Table 5:** Regression analysis of empathy in moderation effect.

Independent Variable: Empathy						
Dependent Variable	Estimate	S.E.	Est./S.E.	*p*-Value	Lower Bound	Upper Bound
Interpersonal relationship	0.251	0.037	6.839	0.000	0.193	0.314
Social responsibility	0.243	0.048	5.029	0.000	0.159	0.319
Interaction 1	0.014	0.003	4.921	0.000	0.009	0.019

**Table 6:** Regression analysis of altruistic behavior in moderation effect.

Independent Variable: Altruistic Behavior						
Dependent Variable	Estimate	S.E.	Est./S.E.	*p*-Value	Lower Bound	Upper Bound
Interpersonal relationship	0.333	0.067	4.997	0.000	0.221	0.333
Social responsibility	0.172	0.092	1.864	0.062	0.024	0.172
Empathy	0.256	0.103	2.488	0.013	0.091	0.256
Interaction 2	0.013	0.006	2.151	0.031	0.003	0.013

**Table 7:** Direct and indirect effects under different values of social responsibility.

	Social Responsibility	Estimate	S.E.	Est./S.E.	*p*-Value	Lower Bound	Upper Bound
Indirect effect	Low value	0.026	0.018	1.44	0.150	0.005	0.069
	Medium value	0.064	0.029	2.249	0.024	0.023	0.116
	High value	0.102	0.044	2.305	0.021	0.036	0.18
Direct effect	Low value	0.202	0.095	2.121	0.034	0.049	0.363
	Medium value	0.333	0.067	4.997	0.000	0.221	0.441
	High value	0.463	0.085	5.471	0.000	0.33	0.61
Total effect	Low value	0.228	0.097	2.363	0.018	0.075	0.397
	Medium value	0.397	0.066	6.056	0.000	0.286	0.504
	High value	0.565	0.076	7.401	0.000	0.437	0.688

**Table 8:** Indirect regulatory effects.

Indirect Regulatory Effect	Estimate	S.E.	Est./S.E.	*p*-Value	Lower Bound	Upper Bound
Interaction 1 × mpathy	0.004	0.002	2.07	0.038	0.001	0.007

## Data Availability

The original contributions presented in the study are included in the article; further inquiries can be directed to the corresponding author.
